# Assessing Dissimilarity Measures for Sample-Based Hierarchical Clustering of RNA Sequencing Data Using Plasmode Datasets

**DOI:** 10.1371/journal.pone.0132310

**Published:** 2015-07-10

**Authors:** Pablo D. Reeb, Sergio J. Bramardi, Juan P. Steibel

**Affiliations:** 1 Department of Fisheries and Wildlife, Michigan State University, East Lansing, Michigan, United States of America; 2 Department of Statistics, Universidad Nacional del Comahue, Cinco Saltos, Rio Negro, Argentina; 3 College of Agricultural and Forest Sciences, Universidad Nacional de La Plata, La Plata, Buenos Aires, Argentina; 4 Department of Animal Science, Michigan State University, East Lansing, Michigan, United States of America; Georgia Institute of Technology, UNITED STATES

## Abstract

Sample- and gene- based hierarchical cluster analyses have been widely adopted as tools for exploring gene expression data in high-throughput experiments. Gene expression values (read counts) generated by RNA sequencing technology (RNA-seq) are discrete variables with special statistical properties, such as over-dispersion and right-skewness. Additionally, read counts are subject to technology artifacts as differences in sequencing depth. This possesses a challenge to finding distance measures suitable for hierarchical clustering. Normalization and transformation procedures have been proposed to favor the use of Euclidean and correlation based distances. Additionally, novel model-based dissimilarities that account for RNA-seq data characteristics have also been proposed. Adequacy of dissimilarity measures has been assessed using parametric simulations or exemplar datasets that may limit the scope of the conclusions. Here, we propose the simulation of realistic conditions through creation of plasmode datasets, to assess the adequacy of dissimilarity measures for sample-based hierarchical clustering of RNA-seq data. Consistent results were obtained using plasmode datasets based on RNA-seq experiments conducted under widely different conditions. Dissimilarity measures based on Euclidean distance that only considered data normalization or data standardization were not reliable to represent the expected hierarchical structure. Conversely, using either a Poisson-based dissimilarity or a rank correlation based dissimilarity or an appropriate data transformation, resulted in dendrograms that resemble the expected hierarchical structure. Plasmode datasets can be generated for a wide range of scenarios upon which dissimilarity measures can be evaluated for sample-based hierarchical clustering analysis. We showed different ways of generating such plasmodes and applied them to the problem of selecting a suitable dissimilarity measure. We report several measures that are satisfactory and the choice of a particular measure may rely on the availability on the software pipeline of preference.

## Introduction

Hierarchical cluster analysis has been a popular method for finding patterns in data and for representing results of gene expression analysis [1]. Clustering algorithms have been widely studied for analyzing microarray data [2,3], however, such technology is being rapidly replaced by RNA sequencing technology (RNA-seq) [4]. In contrast to microarray experiments, RNA-seq generates count data of discrete nature that may call for different analysis methods. One of the most obvious differences between clustering gene expression data from RNA-seq or microarray is the choice of a dissimilarity measure, or the need to transform and normalize RNA-seq data in order to use dissimilarity measures commonly used for microarray data [1].

Before implementing any statistical analysis of RNA-seq data, normalization and transformation have to be performed. [1,5,6]. Normalization aims at reducing non-systematic variation within and between samples, such as sequencing depth and library preparation. Data transformation could be very important because it aims at reducing the effects of skewness, scale and presence of outliers that can be found in read count data that usually follow a Poisson [7] or negative binomial distribution [8,9]. Through appropriate transformation, dissimilarity measures that are sensitive to asymmetric distributions and scale magnitude, such as Euclidean and 1 –Pearson correlation [1,2,10] could be used for clustering RNA-seq data.

Although a Gaussian distribution assumption is not required to compute Euclidean and correlation based distances, transformations that convert count data into a continuous and almost Gaussianly distributed variable [6] could be used for hierarchical clustering. For instance, besides the classical logarithmic transformation, several functions have been proposed to model the mean-variance relationship of RNA-seq data [6,9,11], while accounting for over-dispersion. But the properties of those transformations need to be tested.

Finally, instead of using transformations to approximate the data to a pre-specified distribution where available dissimilarity measures perform well, model based methods can be directly used to compute dissimilarity measures [12].

Evaluating the adequacy of alternative dissimilarity measures for hierarchical clustering requires the fundamental step of choosing reference datasets [13]. An ideal reference dataset should mimic the technical and biological variability found in experimental data, and it should also have some *a priori* known structure in order to assess the goodness of results from alternative analyses. Parametric simulations, exemplar datasets, and permutation sampling have been used to generate such datasets in clustering analysis of biological data [14]. Similarly, plasmode datasets [15] have been proposed for evaluating differential expression analysis in RNA-seq experiments [16]. A plasmode is a dataset obtained from experimental data from which some truth is known, thus, it is an ideal way to generate data with an a priori defined structure that realistically mimics RNA-seq data. Plasmodes were originally proposed for assessing multivariate analysis methods [17] and have been used in behavioral science [18] and also in genomics [19,20].

In this paper, we propose the use of plasmode datasets to assess the properties of dissimilarity measures for agglomerative hierarchical clustering or RNA-seq data. We present two possible ways of creating plasmode datasets that depend on the available data structure, and we use the resulting reference datasets to compare several commonly used dissimilarity measures.

## Materials and Methods

### Datasets

Two experimental datasets were used in this study to create reference datasets. The first dataset, “Bottomly”, corresponds to an experiment described elsewhere [21]. Briefly, 21 samples of *striatum* tissue from two inbred mouse strains (C57BL/6J (B6), n = 10; and DBA/2J (D2), n = 11) were sequenced in three Illumina GAIIx flowcells. Data were downloaded from ReCount website [22]. After filtering out genes with zero counts in all samples, the count matrix contained 13932 rows (transcripts) and 21 columns (samples). The second dataset, “MSUPRP”, corresponds to 24 samples of *longissimus* muscle selected from the MSU Pig Resource Population [23] and sequenced by our collaborators [24]. Total RNA from 24 F2 female pigs of Duroc by Pietrain ancestry was barcoded and sequenced on Illumnina HiSeq 2000. Read mapping, gene modelling and read counting were performed using Tophat [25], Cufflinks [26] and HTSeq [27], respectively. After processing the sequence reads, we obtained a count matrix with 26740 rows (transcripts) and 24 columns (samples). (For details, see file S1 Text). The count matrix of the five samples (animals) used in this paper is available as supporting information in S1 File.

### Plasmodes

Plasmodes are synthetic datasets generated from experimental data for which some true characteristic is known [15]. For instance, we may know *a priori* which genes are not differentially expressed or we may know group membership of each sample. Then, we build a plasmode by re-shuffling the existing data without assuming any probability distributions or correlation structures. Thus, we can use the known characteristic of the synthetic dataset to assess properties of analysis methods. For instance we can apply resampling-based methods to create plasmodes consistent with the null hypothesis (no differential expression) and use them to evaluate the type I error rate hypothesis of testing procedures [28], or we can use the known group memberships to assess the accuracy of clustering methods, as we do in this paper. Thus, plasmodes need to be constructed according to the validation objectives (i.e. considering the statistical method that is being evaluated) and considering the available experimental data.

In this paper, we present two examples on how to create plasmodes to assess the effect of choice of dissimilarity measures on the results of hierarchical clustering of RNA-seq data. In the first experimental dataset, the natural structure of the data is known *a priori* and it was generated through the experimental design (sequencing flowcells and mice strains), while in the second experimental dataset there is not an *a priori* known structure, so we create a set of artificial samples where the structure is generated by construction.

### Plasmodes from Bottomly dataset

**Fig 1:**
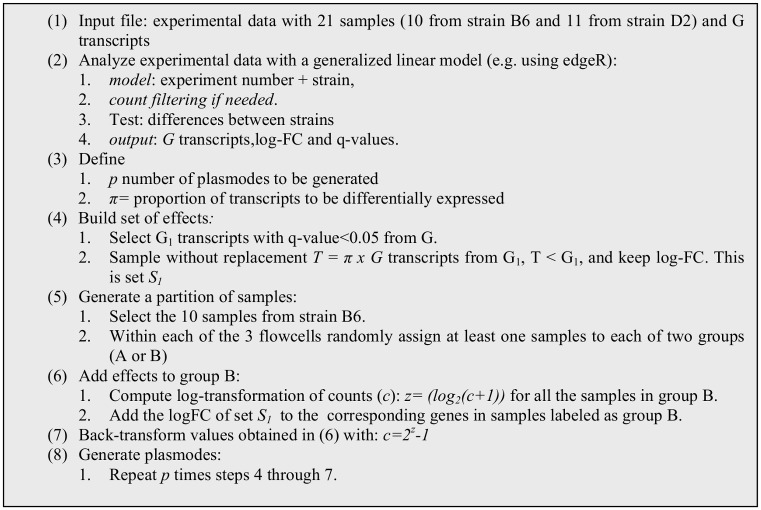
Algorithm used to generate plasmodes from Bottomly dataset.

We built plasmodes for this dataset by using samples from B6 strain, partitioning them in two groups and adding known effects for selected genes taken from the difference in gene expression with strain D2. Fig 1 presents the algorithmic steps used to generate the plasmodes. Two main effects, strain and flowcell, were used to classify the 21 samples (Step 2.1 in Fig 1) given the importance of both sources of variation has been described before [6,16]. Then, a differential expression analysis including all the samples (both strains) was conducted with edgeR [8] and transcripts with q-value <0.05 were identified as differentially expressed (set *G*_*1*_ in step 4 of Fig 1). Subsequently, samples from strain B6 were randomly assigned to two groups (A or B) within each flowcell, and a subset (*S*_*1*_) of effects randomly selected from *G*_*1*_ was added to the corresponding genes in samples labelled as B (Steps 5–6). Therefore, samples from group A and B differ due to the strain effect added by the subset (*S*_*1*_) of differentially expressed genes, while samples within each group differ due to the flowcell effect. We generated 50 plasmodes with 10% of differentially expressed transcripts by defining p = 50 and π = 0.10 in step 3 and by randomly assigning 2 samples to group B and one or two samples, if available, to group A within each flowcell (Step 5.2 in Fig 1). As a result, in each plasmode generation we obtained a total of 10 samples under two artificial treatments (A or B) and three flowcell effects (1, 2 or 3), resulting in a set of samples indexed by such factors as:{(A_1_, A_1_, B_1_, B_1_),(A_2_,B_2_,B_2)_,(A_3_,B_3_,B_3_)}. If we use only differentially expressed genes, we expect the samples with same letter to cluster together because of the treatment effect, but as we add a large number of non differentially expressed genes, we can expect that samples with the same subindex (flowcell) will tend to cluster together because it has been shown before that there is a strong flowcell effect in this experiment [6,16]. To evaluate the performance of dissimilarity measures under various differentially expressed / non differentially expressed ratios (DE/nonDE), we analyzed three scenarios for each plasmode: 1) only DE transcripts (DE_[100%]_), 2) DE transcripts + all nonDE transcripts (DE_[10%]_+nonDE_[90%]_), and 3) DE transcripts + a random sample of 50% from nonDE transcripts (DE_[20%]_+nonDE_[80%]_).

### Plasmodes from MSUPRP dataset

**Fig 2:**
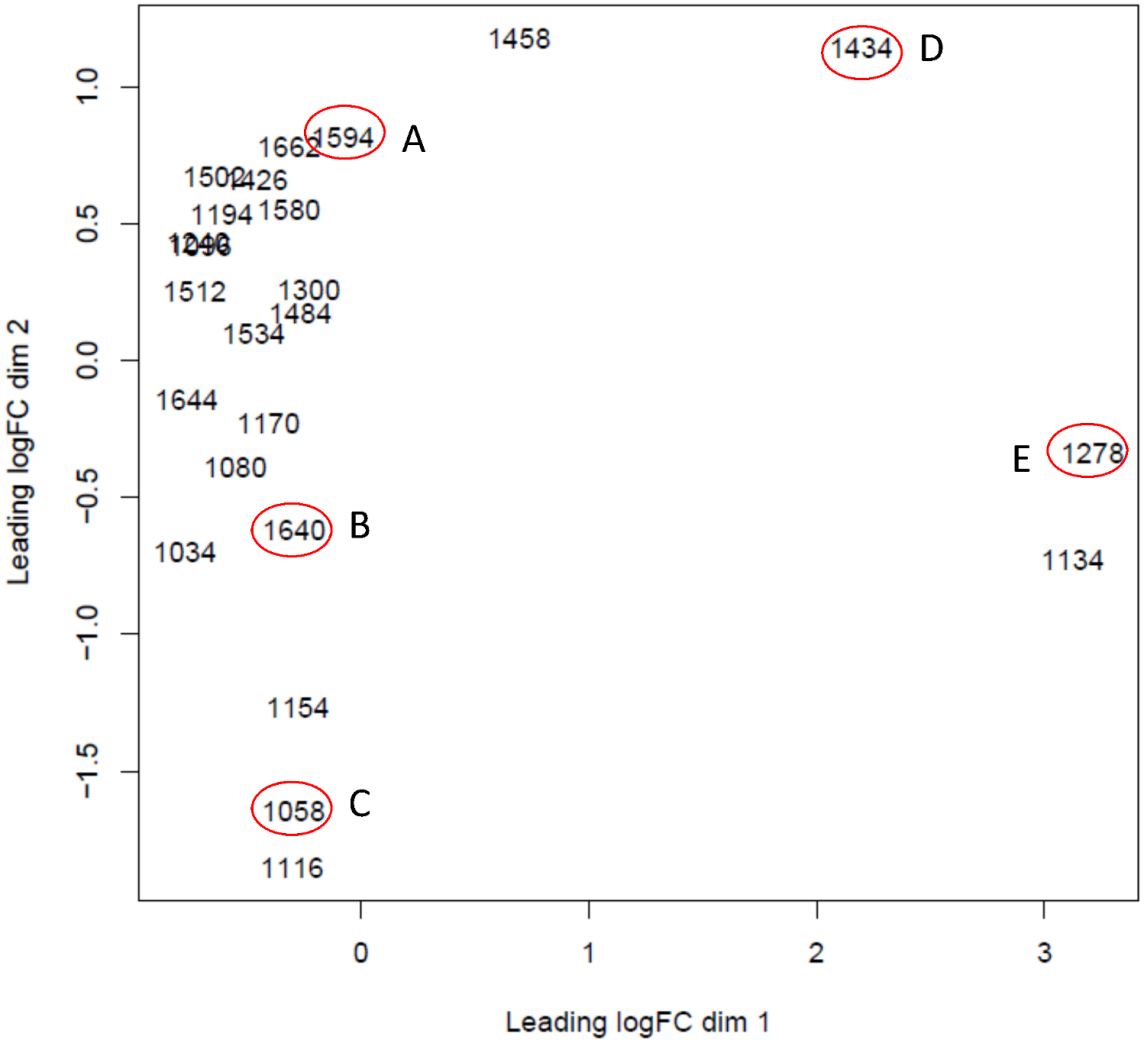
Multidimensional scaling analysis of MSUPRP dataset. Twenty four samples were represented in the main plane (dimension 1 and dimension 2 explained 22.4% and 13.8% respectively) and five distant samples (A, B, C, D, E, marked with ovals) were selected as input samples to generate plasmode datasets.

Since this dataset did not have a natural sample structure derived from experimental conditions, a structure had to be induced in order to know a priori the expected clustering configuration. From a descriptive multidimensional scaling analysis of the 24 pig samples (animals), we selected 5 dissimilar samples (A, B, C, D, E) according to their configuration in the main plane (Fig 2). Synthetic samples were generated by combining a known proportion of randomly sampled read counts of individual genes from each of two of the five selected samples. For instance, a new synthetic sample named AAC was generated combining 2/3 and 1/3 of read counts of individual genes from A and C respectively. A full plasmode consisted of 12 samples that included the five selected samples {AAA, BBB, CCC, DDD, EEE}, five synthetic samples {AAC, BBC, CCB, DDE, EED} obtained by combining 2/3:1/3 proportions from two of the selected samples, and two synthetic samples {CxB, ExD} obtained by combining 1/2:1/2 proportions of two of the selected samples (see S2 Fig in S2 Text with a representation of the relationships among the 12 samples of each plasmode). Following this procedure, a total of 50 replicated plasmodes were generated. As a result we created a synthetic dataset where the samples were expected to resemble each other to a known degree given the proportions of shared reads.

### Clustering

Defining a dissimilarity measure and a linkage method are the two key decisions for performing hierarchical cluster analysis. We focused on assessing the adequacy of dissimilarity measures that have been commonly used for clustering gene expression data. We also include a recently proposed dissimilarity measure for RNA-seq count data [12]. As linkage method, we decided to use complete linkage because it is invariant under monotone transformations [29], and hence dissimilarity measures that have the same relative ranking result in the same cluster structure [1]. This robustness reduces the effect of linkage method when comparing dendrograms and allowed us to concentrate in the evaluation of dissimilarity measures. Hierarchical cluster analysis was applied to each plasmode using the agglomerative procedure implemented in function hclust from R [30] to concatenate samples and to generate dendrograms.

Eight dissimilarity measures were compared, including 4 variants based on Euclidean distance, 3 correlation based approaches, and one Poisson based measure. Euclidean distances were computed between samples following one of 4 approaches: i) using raw count data (*raw*), ii) after normalizing samples using the median ratio size factor proposed by Anders and Huber [9] (*rnr*), iii) after applying a variance stabilizing transformation computed with DESeq2 [31] (*vsd*), and iv) after applying a regularized logarithm transformation implemented in DESeq2 [31] (*rld*). Correlation based dissimilarities comprised: i) 1- Pearson correlation between samples using raw counts (*pea*), ii) 1- Pearson correlation between samples using counts transformed by logarithm of raw counts +1 (*plg*), and iii) 1- Spearman correlation between samples using raw counts (*spe*). The Poisson dissimilarity (*poi*), which is based on a log likelihood ratio statistic for a Poisson model [12], was computed on data that were transformed by a power function to account for overdispersion, and normalized by total sum of counts for each sample.

### Cluster validation using results from plasmodes

Cluster validation can be assessed using several indices [32,33] and the choice of a particular measure is application dependent [2]. Cophenetic distances provide a way to quantify similarities among dendrograms in hierarchical clustering. The cophenetic distance is the distance from the bottom of the tree at which two elements (samples in this paper) are grouped in the same cluster for the first time in the hierarchy. To represent a dendrogram in terms of a set of cophenetic distances, the distances between all pairs of elements is computed and arranged into a matrix called cophenetic matrix that represents the whole hierarchy, as illustrated in S5 and S6 Figs in S2 Text. Cophenetic matrices can be used to compare dendrograms [34]. For instance, to compare how similar are two dendrograms, the Pearson correlation between the lower triangular portions of two cophenetic matrices can be used.

We computed the correlation between cophenetic matrices [13] to compare dendrograms obtained with different dissimilarity measures (between dissimilarity measure comparison) as well as to compare all dendrograms obtained with a particular dissimilarity measure (within dissimilarity measure comparison). Mean and standard deviation of correlations between dissimilarity measures were used as a measure of agreement while mean and standard deviation of correlations within a dissimilarity measure were used as a measure of consistency.

We also visually compared the obtained dendrograms to a reference dendrogram built according to the sample structure known *a priori* from the plasmode generation process in the MSUPRP dataset. For the MSUPRP dataset, we defined the expected similarity between two samples (*s*_*ij*_) as the maximum proportion of shared reads, and we defined 1- *s*_*ij*_ as a reference dissimilarity (see S3 Fig in S2 Text). With the correlation between each of the dissimilarity matrices and the reference dissimilarity, we assessed how well each dissimilarity measure recovered the expected sample structure. An equivalent reference dissimilarity matrix and reference dendrogram cannot be easily built for the Bottomly dataset because we did not exploit relationships between samples to build the plasmode, except for their group membership. In this case, we compared typical dendrograms obtained from plasmodes to the known strain and experiment membership in the original data.

## Results

### Bottomly

**Fig 3:**
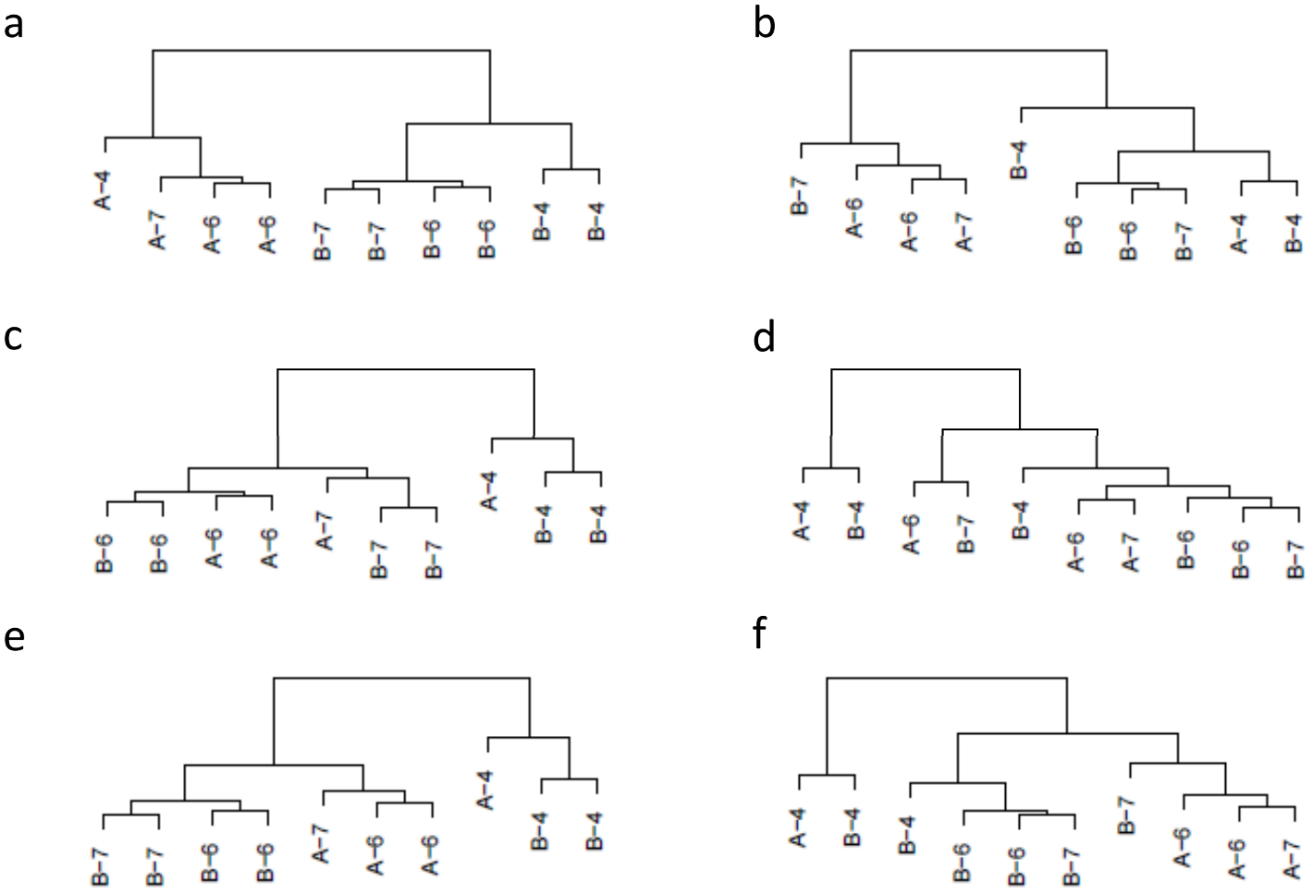
Typical dendrograms obtained for plasmode datasets from Bottomly experimental data with two dissimilarity measures under three scenarios. Dendrograms obtained using complete linkage hierarchical clustering based on Poisson dissimilarity (*poi*) are presented in the left column (a, c and e), and dendrograms based on Euclidean distance calculated from raw normalized data (*rnr*) are presented in right column (b, d, f). The rows correspond to three scenarios with different percentage of differentially expressed (DE) transcripts: 1) DE_[100%]_ (a and b), 2) DE_[10%]_+nonDE_[90%]_ (c and d), and 3) DE_[20%]_+nonDE_[80%]_ (e and f). Sample labels correspond to main treatment (A or B) and flowcell number (4, 6 or 7). Dendrograms based on *poi* separates samples according to the expected sources of variation; in (a), only DE transcripts, samples are arranged in two separate groups following treatment labels; in (c), with a predominant number of non DE transcripts, the structure of groups is dominated by flowcell characteristics in addition to main treatment: and in (e) an in-between scenario, the dendrogram presents an intermediate group structure. Dendrograms based on *rnr* do not resemble any expected configuration.

**Fig 4:**
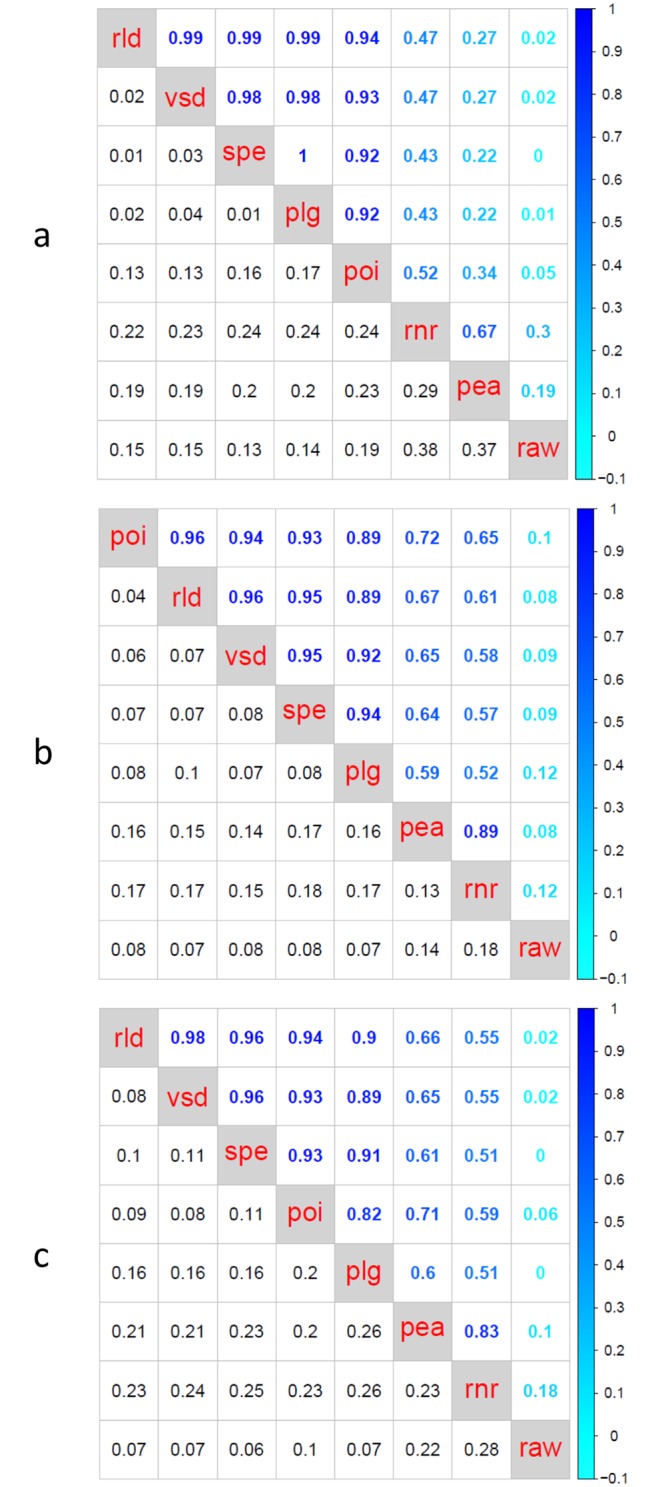
Agreement between dissimilarity measures using Bottomly plasmode datasets. Each matrix contains means (upper triangle) and standard deviations (lower triangle) of correlation between cophenetic matrices of dendrograms (N = 50 plasmode datasets) for eight dissimilarity measures: Euclidean distances using raw count data (*raw*), Euclidean distances using normalized samples (*rnr*), Euclidean distances using variance stabilizing transformation (*vsd*), Euclidean distances using regularized logarithm (*rld*).) 1- Pearson correlation using raw counts (*pea*), 1- Pearson correlation using counts transformed by logarithm of raw counts +1 (plg), and 1- Spearman correlation using raw counts (*spe*), and Poisson dissimilarity (*poi*). Panel labels (a), (b) and (c) correspond to one of three scenarios of proportion of differential expressed genes: DE_[100%]_, DE_[10%]_+nonDE_[90%]_, and DE_[20%]_+nonDE_[80%]_, respectively. In all scenarios, we identified three sets of dissimilarity measures: 1) *raw*, 2) *rnr* and *pea*, and 3) *poi*, *rld*, *vsd*, *plg* and *spe*. Results from *raw*, set 1, were poorly related to results from any other dissimilarity measure. Dendrograms from dissimilarity measures in set 2 presented correlation of cophenetic matrices with medium to high means and high variability with each other, and low correlation with dendrograms from other dissimilarity measures. Dendrograms from dissimilarity measures in set 3 exhibited high correlations of cophenetic matrices and low to medium variability when compared to each other.

Fig 3 shows the typical dendrograms obtained for plasmode datasets using two dissimilarity measures, *poi* and *rnr*, which are representative examples of two sets of results under the three different scenarios (DE_[100%]_, DE_[10%]_+nonDE_[90%]_, and DE_[20%]_+nonDE_[80%]_,). On the one hand, scenario 1 (DE_[100%]_) uses only differentially expressed transcripts, therefore the expected hierarchy should arrange samples in two separate groups according to main treatment labels. Such is the structure obtained utilizing the Poisson (*poi*) dissimilarity measure (Fig 3a). Using the Poisson dissimilarity measure, samples were clustered in two groups corresponding to treatments A or B, and within each of the groups, samples were arranged according to block numbers (4, 6, or 7). Differently, the dendrogram based on Euclidean distance calculated from raw normalized data (*rnr*) (Fig 3b) mixed treatment labels and did not recover any expected structure. On the other hand, scenario 2 (DE_[10%]_+nonDE_[90%]_), uses information from differentially (10%) and non differentially expressed (90%) transcripts. As a result, we expected that the dissimilarity measures would tend to represent other aspects of samples in addition to the treatment effect. In concordance with such expected structure, dendrogram obtained using the Poisson dissimilarity (Fig 3c) firstly separated samples according to block labels, block 4 being the most different group. Subgroups for treatments A and B were arranged within each block. Conversely, dendrogram based on Euclidean distance calculated from raw normalized data (*rnr*) (Fig 3d) did not present any expected structure. Finally, scenario 3 (DE_[20%]_+nonDE_[80%]_) represents an intermediate case that is useful to further explore the performance of dissimilarity measures because it is enriched in DE genes with respect to scenario 1, but it still conserves 80% of background (nonDE) genes. The dendrogram based on the Poisson dissimilarity (Fig 3e) presented an intermediate structure where we observed that samples from block 4 were clustered together while the remaining samples were clustered in a separate group mainly classified by treatment effect. Yet again, dendrogram based on *rnr* (Fig 3f) did not characterize any expected configuration. To sum up, for this dataset, dendrograms generated from a Poisson dissimilarity resemble the expected hierarchical structures in all three scenarios, however, dendrograms based on Euclidean distance computed on raw normalized data did not. Comparison of hierarchies between clusters constructed using *poi* and *rnr* dissimilarities across 50 plasmodes presented correlation of cophenetic matrices with low means and high standard deviations (0.52±0.24, 0.65±0.17, 0.59±0.23, for scenarios 1, 2 and 3, respectively) (Fig 4). These results emphasize a poor correspondence between hierarchies constructed upon *poi* and *rnr* dissimilarities.

Correlations between hierarchies obtained with the eight dissimilarity measure approaches for each of three scenarios (DE_[100%]_, DE_[10%]_+nonDE_[90%]_ and DE_[20%]_+nonDE_[80%]_) are presented in Fig 4. Each matrix contains means and standard deviations of correlations between cophenetic matrices, in the upper and lower triangle respectively. Regardless of the scenario, dissimilarity measures can be apportioned to three groups with common patterns. First, Euclidean distance computed on raw data (*raw*) is poorly related to any other dissimilarity measure. Second, Euclidean distance computed on normalized data (*rnr*) and 1- Pearson correlation dissimilarity (*pea*) presented medium to high correlations of cophenetic matrices (mean from 0.67 to 0.89) with high variability (standard deviation from 0.13 to 0.29) with each other and low correlation values with other dissimilarity measures. Third, there is a subset comprising 1-Pearson correlation dissimilarity computed on log-transformed counts (*plg*), 1-Spearman correlation dissimilarity (*spe*), Euclidean distance computed on transformed counts after applying either a variance stabilizing function (*vsd*) or a regularized logarithm (*rld*), and the Poisson dissimilarity (*poi*). This last group of dissimilarity measures presents high correlations of cophenetic matrices (mean from 0.82 to 0.99) with low to medium variability (standard deviation from 0.01 to 0.2). Only hierarchies obtained with dissimilarity measures from the third group consistently presented the expected natural structure created by design in the plasmode generation process, being *rld*, *vsd*, *spe* and *plg* the more consistent across all scenarios (Table 1, columns 2, 3 and 4).

**Table 1:** Consistency for each dissimilarity measure.

Dissimilarity	Bottomly			MSUPRP
	DE_[100%]_	DE_[10%]_+nonDE_[90%]_	DE_[20%]_+nonDE_[80%]_	
***raw***	0.58 (0.22)	0.91 (0.13)	0.75 (0.20)	0.56 (0.23)
***rnr***	0.35 (0.27)	0.43 (0.28)	0.33 (0.28)	0.40 (0.22)
***rld***	0.98 (0.01)	0.90 (0.11)	0.88 (0.13)	0.99 (0.01)
***vsd***	0.96 (0.04)	0.91 (0.10)	0.86 (0.15)	0.99 (0.01)
***pea***	0.37 (0.31)	0.53 (0.30)	0.45 (0.30)	0.43 (0.28)
***plg***	0.98 (0.01)	0.89 (0.13)	0.75 (0.19)	0.98 (0.01)
***spe***	0.99 (0.01)	0.86 (0.14)	0.88 (0.14)	0.99 (0.01)
***poi***	0.86 (0.21)	0.92 (0.09)	0.88 (0.14)	0.99 (0.01)

Mean and standard deviation of correlation between cophenetic matrices of dendrograms (N = 50 plasmode datasets) for each of eight dissimilarity measures: Euclidean distances using raw count data (*raw*), Euclidean distances using normalized samples (*rnr*), Euclidean distances using regularized logarithm (*rld*) Euclidean distances using variance stabilizing transformation (*vsd*), 1- Pearson correlation using raw counts (*pea*), 1- Pearson correlation using counts transformed by logarithm (plg), and 1- Spearman correlation using raw counts (*spe*), and Poisson dissimilarity (*poi*). Columns correspond to the three scenarios generated for Bottomly (with different proportion of DE genes) and the MSUPRP dataset. We considered a clustering from a dissimilarity measure to be consistent if hierarchies obtained for different plasmode datasets within each dissimilarity measure were highly correlated and presented a low standard deviation. Clustering based on *raw*, *rnr* and *pea* were generally inconsistent presenting a number of very different hierarchical structures.

We considered a dissimilarity measure to be consistent if hierarchies obtained for different plasmode datasets within each dissimilarity measure were highly correlated and presented a low standard deviation. Consequently, we computed correlations of cophenetic matrices for dendrogram within each dissimilarity measure and calculated the mean and standard deviation for each ensemble of 50 plasmodes (Table 1, columns 2, 3 and 4). Dissimilarity measures *raw*, *rnr* and *pea* were generally inconsistent, resulting in a number of different hierarchical structures. For instance, *rnr* presented mean correlation values of 0.35±0.27, 0.43±0.28, and 0.33±0.28 for the three respective scenarios. Conversely, all the other dissimilarity measures were much more consistent. For example, *rld* presented mean correlation values of 0.98±0.01, 0.90±0.11, and 0.88±0.13 for the three respective scenarios. Such high values mean that hierarchies obtained with *rld* for the 50 plasmodes were all very similar to each other.

### MSUPRP

**Fig 5:**
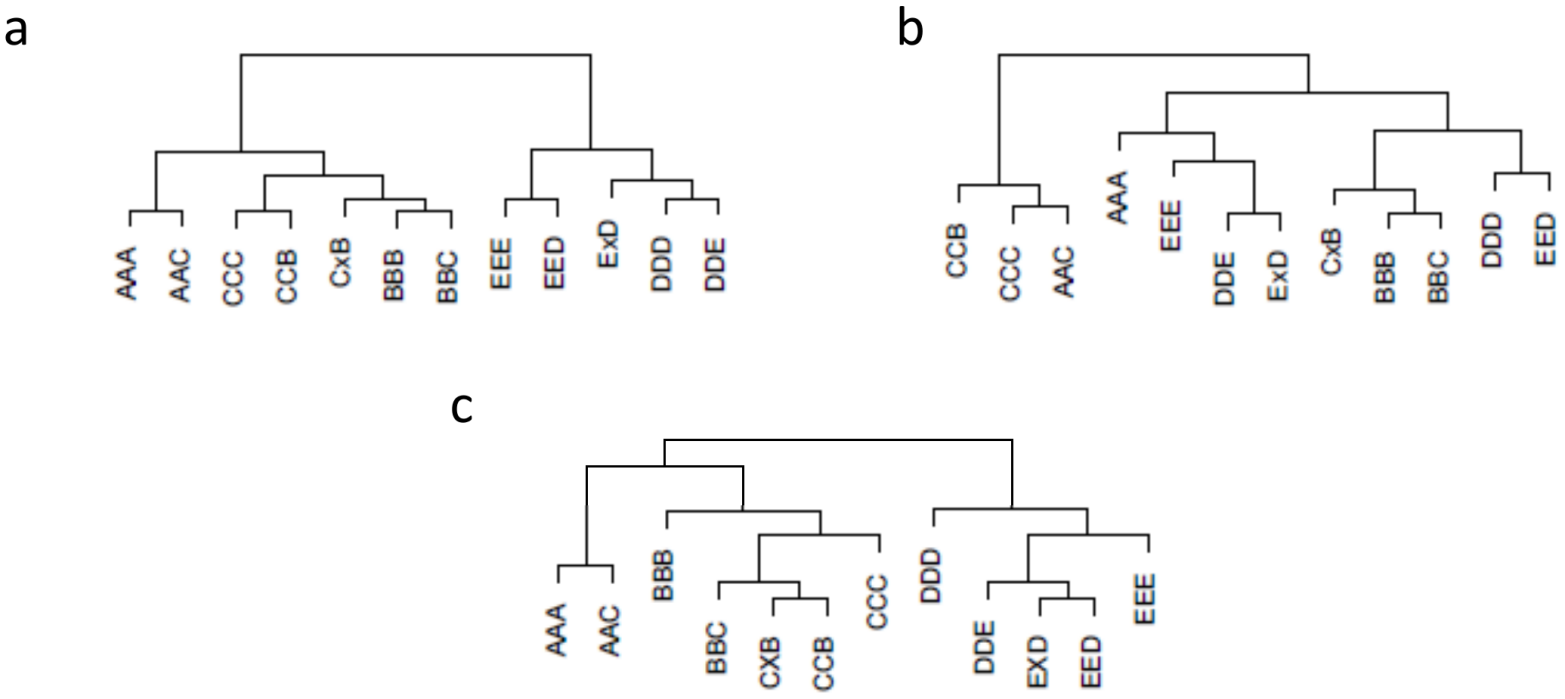
Typical dendrograms obtained for plasmode datasets from MSUPRP experimental data with two dissimilarity measures. Dendrograms using complete linkage based on (a) Poisson dissimilarity (*poi*), (b) Euclidean distance calculated from raw normalized data (*rnr*), and (c) reference dissimilarity based on maximum proportion of shared reads. Original samples are labeled with 3 same letters (AAA, BBB, CCC, DDD or DDD), synthetic samples are labelled with 2 or 3 letters symbolizing the proportion of transcripts, ½ or ⅓ respectively, taken from the original samples. Dendrogram (a) clustered original samples A, B and C and their synthetic combinations in one group, and original samples E and D and their synthetic combinations in another group. The hierarchical structure of each of these two groups represented the degree of shared reads between samples by joining first samples that shared ⅓ of reads and then samples that shared ½ of reads. Dendrogram (b) did not cluster samples according to the expected configuration.

**Fig 6:**
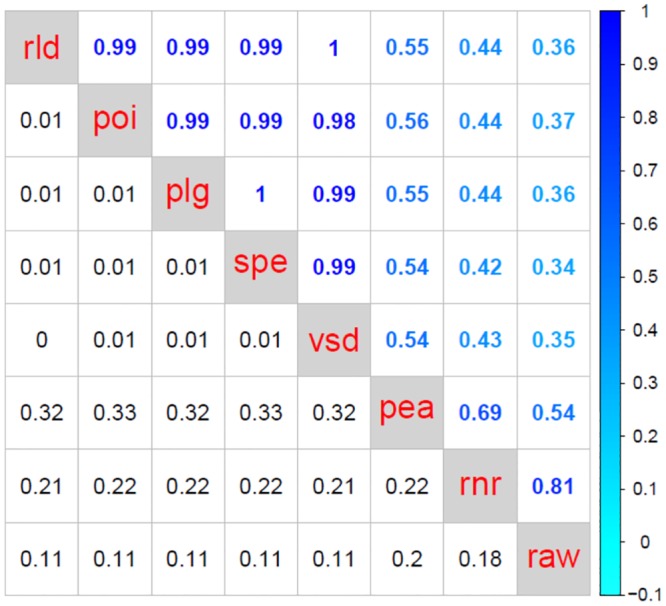
Agreement between dissimilarity measures using MSUPRP plasmode datasets. The matrix contains means (upper triangle) and standard deviations (lower triangle) of correlation between cophenetic matrices of dendrograms (N = 50 plasmode datasets) for eight dissimilarity measures: Euclidean distances using raw count data (*raw*), Euclidean distances using normalized samples (*rnr*), Euclidean distances using variance stabilizing transformation (*vsd*), Euclidean distances using regularized logarithm (*rld)* 1- Pearson correlation using raw counts (*pea*), 1- Pearson correlation using counts transformed by logarithm of raw counts +1 (*plg*), and 1- Spearman correlation using raw counts (*spe*). We identified the same three sets of dissimilarity measures described before: 1) *raw*, 2) *rnr* and *pea*, and 3) *poi*, *rld*, *vsd*, *plg* and *spe*.

Plasmodes from MSUPRP were constructed by combining known proportions of sequence reads from pairs of samples, including nonDE as well as potentially DE transcripts across individual. We expect that dendrograms cluster the samples according to the known proportions of shared reads as presented in the reference dendrogram in Fig 5c (see S3 Fig in S2 Text with the corresponding reference dissimilarity matrix). Fig 5a and 5b present the typical dendrograms obtained for plasmode datasets using *rnr* and *poi*, which are representative examples of the 8 dissimilarity metrics. Dendrogram based on the Poisson dissimilarity (Fig 5a) clustered the original samples A, B and C and their synthetic combinations in one group, and original samples E and D and their synthetic combinations in a distinct group. The hierarchical structure of each of these two groups represented the degree of shared reads between samples by joining first samples that shared ⅓ of reads and then samples that shared ½ of reads. Additionally, the separation between samples {A, B, C} and {D,E} agreed with positions along the most important dimension (dim1) in Fig 2. In contrast, dendrogram based on *rnr* (Fig 5b) did not cluster samples according to the anticipated configuration. Comparison of hierarchies between clusters constructed from *rnr* and *poi* dissimilarities for all plasmodes presented low mean and high standard deviations (0.44±0.22) of correlation between cophenetic matrices (Fig 6).

Correlations between hierarchies for the eight dissimilarity measures are summarized in Fig 6. It contains mean and standard deviations, in the upper and lower triangle respectively, of correlations between cophenetic matrices computed on 50 plasmode datasets. As observed in the three scenarios for the Bottomly experiment, dissimilarity measures can be apportioned to three groups: 1) *raw*, 2) *rnr* and *pea*, and 3) *poi*, *rld*, *vsd*, *plg* and *spe*. Dendrograms from *raw* did not agree with dendrograms from other groups. Hierarchies from dissimilarity measures in group 2 presented a medium correlation of cophenetic matrices with high variability (0.69±0.22). Dendrograms from the dissimilarity measures in group 3 presented high correlation values with each other (>0.98) and low variation (<0.01).

The correlation of hierarchies within each of the dissimilarity measures (Table 1, column 5) was low for *raw*, *rnr* and *pea*, whereas clusters were much more consistent (*r*>0.98) for *poi*, *rld*, *vsd*, *plg* and *spe* dissimilarities. Additionally, dissimilarity measures *raw*, *rnr*, and *pea* were poorly correlated with the reference dissimilarity (0.57±0.07, 0.53±0.07, and 0.51±0.04, respectively) while *poi*, *rld*, *vsd*, *plg*, and *spe* were highly correlated with the reference dissimilarity (r>0.8±0.001, see S1 Table in S2 Text). Consequently, dissimilarities raw, *rnr*, and *pea* did not resemble the expected sample structure and resulted in dendrograms that were very inconsistent over repeated sampling of the same dataset. In contrast, dissimilarities *poi*, *rld*, *vsd*, *plg*, and *spe* maintained the sample structure and produced highly reproducible results in hierarchical dendrograms.

## Discussion

Hierarchical cluster analysis is one of the most used techniques for exploring expression patterns in sequencing data [1]. In this paper, we showed how to assess the adequacy of dissimilarity measures for clustering samples from RNA-seq experiments by generating plasmode datasets from experimental data.

Plasmode datasets are useful alternatives to parametric simulations for assessing statistical methodologies as data are generated on more realistic conditions and do not depend on a specific parametric model [19]. The algorithm used to build a plasmode dataset depends on the characteristics of the experimental data and the objective of the study. We presented two examples on how to build plasmode datasets from two experiments with different conditions.

The Bottomly dataset had an experimental design with two main sources of variation and used highly inbred individuals. Such context allowed us to generate plasmode datasets with known proportions of differentially expressed transcripts (Fig 1) and focused on assessing the adequacy of dissimilarity measures in recovering the main sources of variation in the hierarchical structure (Fig 3). Analogous plasmode generation algorithms have been used with different objectives, for example to validate differential expression methods for RNA-seq [16], microarray analysis[19], and qPCR [20], but this is the first time that they are used to assess the properties of sample-based clustering. These procedures are by no means exhaustive of the possible ways of creating plasmodes for clustering. For instance, the algorithm presented in Fig 1 preserves the correlation among genes [16] when generating plasmodes, but other sampling strategies could purposefully select groups of genes with specific correlation patterns. For instance, instead of sampling from DE and nonDE groups, transcripts could be sampled from blocks of co-expressed genes, resulting in more realistic datasets especially if the study is focused on gene-based clustering and co-expression analysis [35,36].

Different from the Bottomly dataset, the MSUPRP did not present any experimental treatment, and individual characteristics were more important. Under these circumstances, we built plasmodes creating synthetic individuals, by combining known proportions of read counts from original individuals and we evaluated the adequacy of dissimilarity measures in resembling the different mixture proportions in the hierarchical sample structure (Fig 5). A similar plasmode generation algorithm was proposed [37] to evaluate admixture estimation methodologies where the objective is to estimate the proportion of an individual’s genome that originates from different founding populations, but using SNP genotypes instead of sequence read counts.

Although the utility of plasmode datasets has been recently highlighted in RNA-seq studies [16,38], only parametric simulations or exemplar experimental datasets have been used to compare dissimilarity measures and clustering methods [12,39]. While extremely useful, parametric simulations are often criticized as being too simplistic to appropriately capture the complexity in gene expression data [40], thus limiting the scope and validity of the resulting conclusions. On the other hand, using a single exemplar dataset with unknown properties is not an appropriate approach for comparing statistical methods [15]. As a partial solution to these limitations, in this paper we show that plasmodes can supplement the evaluation of clustering algorithms by including agreement and consistency measures based on datasets that mimic read count distributions more realistically. One likely criticism of plasmode is that the results may heavily depend on the original dataset [16]. However, this does not invalidate their use. Moreover, as shown in this paper, using two very different datasets, some properties of alternative metrics remain consistent, which encourages the use of the plasmode generation methods presented here using alternative datasets.

We built plasmodes to evaluate alternative dissimilarity measures. The selected dissimilarity measures allowed i) the comparison of traditional dissimilarity measures and dissimilarity measures based on discrete count distributions specifically proposed for RNA-seq, and ii) studying the effect of normalization and transformation prior to computing dissimilarities.

The Euclidean distance or the Pearson correlation based dissimilarity computed after transforming data is a routine method adopted from microarray gene expression analysis [1,2]. In fact, the Pearson correlation based dissimilarity is equivalent to squared Euclidean distance of standardized data [41]. On the other hand, the most common transformations used for RNA-seq are the logarithm of counts, or logarithm of counts plus a constant [42], but other variance stabilizing and regularized logarithm transformation functions have been proposed to model the mean-variance relationship of RNA-seq counts [6,9,31]. The Spearman correlation based dissimilarity uses the rank of the read count instead of the counts themselves to compute correlation; consequently it could be applied without transforming the data. Although the use of Spearman correlation based dissimilarity has been discouraged for gene-based clustering of RNA-seq data [1], because it uses a small number of grouped samples to compute ranks, we have used it for sample-based clustering where the number of genes is potentially large enough to obtain more precise ranks. Finally, the Poisson dissimilarity [12] was specifically proposed for clustering of sequencing data based on a Poisson log-linear model of normalized counts, and thus, it is a natural candidate to be included in this comparison.

The eight evaluated dissimilarity measures presented a common pattern of agreement and consistency in recovering the expected sample structure for both plasmode datasets. Dissimilarity measures with high level of agreement between them—correlations between cophenetic matrices with high mean and low standard deviation (Figs 4 and 6)—produce dendrograms with very similar hierarchical structures. However, if dissimilarity measures have correlations of cophenetic matrices with either low mean or high standard deviation (Figs 4 and 6), they generate dendrograms with different hierarchical structures. In addition, correlation between dendrograms obtained with a particular dissimilarity measure summarizes the consistency of such dissimilarity measure. If a dissimilarity measure has a within cophenetic correlation with high mean and low standard deviation, it consistently generates similar dendrograms.

To assess the adequacy of a dissimilarity measure, both agreement and consistency are important. We showed this with *poi*, *rld*, *vsd*, *plg* and *spe* dissimilarities, which presented similar level of agreement in both datasets (Figs 4 and 6). However, dendrograms based on these dissimilarity measures were consistent in the MSUPRP dataset, but showed different consistency under the three scenarios in the Bottomly datasets (Table 1). A counter example is dissimilarity measures *rnr* and *pea* that agreed with each other but were very inconsistent. This means that *rnr* and *pea* tended to reproduce similar clusters on each plasmode, and wide range of dendrograms structures. This has not been reported before, because consistency of clustering under repeated sampling has not been studied in previous works. But a reason for the agreement is that both *rnr* and *pea* are focusing on the same features, because they are essentially normalized Euclidean distances. The reason for the low consistency could be that these measures are expected to behave well with heterogeneous approximately Gaussian data, but not too well with extremely non-Gaussian data. On the other side, the measure *raw* is expected to be better suited for homogenous Gaussian data (all variances are of similar magnitude).

As mentioned before, we used plasmode to study the effect of normalizing data. Normalization is an essential data processing step in RNA-seq analysis that aims at removing systematic biases in order to make consistent comparisons within and between samples[43]. Although several methods[5,9,44] have been proposed to normalize data, especially for differential expression analysis, the impact of a particular normalization method seemed to be less important in classification and clustering analyses [12]. We confirmed this, showing that normalizing counts to equal library sizes was not enough to capture the natural structure of samples when it was the only transformation applied. For instance, dissimilarity measures *raw* and *rnr* had low agreement with dissimilarity measures that resemble better the true structure of data, e.g. *rld*, *vsd*, *spe* or *plg* or *poi* (Figs 4 and 6).

Accounting for the discrete nature of read counts in RNA-seq data is the most important issue to consider when computing dissimilarity measures. For instance, Euclidean distance and Pearson correlation based measures are known to be influenced by scale, skewness and outliers, thus, they may not work well for count data [1]. In support of this, we found that dissimilarity measures *raw*, *rnr*, *pea*, based directly on counts, regardless of normalization or standardization, did not resemble the expected dendrogram and were generally inconsistent. Dendrograms obtained with Pearson based correlation resembled the expected structures only when data were previously log-transformed. However, we found that the Spearman correlation based dissimilarity measure (*spe*) was suitable to represent the natural structure of samples even without normalizing data, possibly because it preserves the relative rank relationships, and it is less influenced by skewness and outliers [45] when it is based on a large number of genes. The variance stabilizing (*vsd*) and regularized logarithm (*rld*) approaches consistently retrieved the expected dendrogram structure. Both transformations model the mean-variance relationship across all genes to stabilize the variance of counts across samples [9,31]. The regularized logarithm transformation also accounts for variation in sequencing depth across samples [31]. Both functions have been suggested as appropriate transformations for clustering and classification of RNA-seq data with less ambiguous results in hierarchical clustering than using simply log-transformed counts [31]. Finally, directly using the Poisson dissimilarity (*poi*) generated dendrograms with the expected structure. This is not surprising considering that read counts are usually assumed to fit over dispersed Poisson distributions[46]. Similarly, Witten [12] obtained dendrograms with lower clustering error rates when using the Poisson dissimilarity rather than *vsd* or Euclidean distances on normalized data, but using overdispersed Poisson simulations. Our results are encouraging because we did not use a parametric model to produce similar outcomes.

Sample-based hierarchical cluster analysis can be used as a tool to present results after differential expression analysis or it can be used as an explorative technique for finding patterns in data. In the first approach only informative genes, i.e. differentially expressed genes (called signal in data mining literature) are used while in the second approach informative as well as non informative genes (also known as noise) are utilized [2]. As the signal-to-noise ratio (proportion of DE to nonDE genes) is usually less than 1:10 [2], particular methods are applied to diminish the influence of non informative genes that can degrade the reliability of clustering results [2]. In RNA-seq analysis, cluster analysis is commonly applied only to differentially expressed genes or a subset of them [1]. We have assessed dissimilarity measures under scenarios that include not only a set of differentially expressed transcripts but we also combined differentially and non differentially expressed transcripts (signal-to-noise ratio 1:9 and 1:4), as well as a mixture of individuals. We found that *rld*, *vsd*, *plg*, *spe* and *poi* were highly consistent under all scenarios with a tendency to diminish consistency as the number of non informative genes increases. Although we focused our comparison on the effect of dissimilarity measures on hierarchical clustering results, the same plasmodes could be used to investigate the effect of other decisions made when performing sample-based clustering as the selection of the hierarchical clustering algorithm per-se or even the effect of pre-filtering transcript according to their level of expression [47–49]. We did not explore those aspects of sample clustering, but their investigation will be facilitated by the plasmode building strategies described in this paper.

## Conclusions

Generating plasmode datasets from experimental data is a reliable tool for evaluating dissimilarity measures in agglomerative hierarchical cluster analysis of RNA-seq data. Depending on the characteristics of the available datasets, several scenarios can be established to compare dissimilarity measures upon a broad spectrum of more realistic conditions than using other simulation approaches. Similar methodologies can be applied to study gene-based clustering as well as other clustering analysis methods.

Explorative sample-based hierarchical clustering of RNA-seq data needs as an input a dissimilarity matrix that accounts for the mean-variance relationship of the discrete nature of read counts. Euclidean distance calculated either on data that have been previously logarithm-transformed or regularized with more complex *ad hoc* functions, as well as model-based dissimilarity for RNA-seq data, were consistent in reproducing the expected sample structure in hierarchical dendrograms.

## Supporting Information

S1 File MSUPRP dataset. This file contains raw count data (6 columns x 25798 rows) for the five animals used to generate the MSUPRP plasmode datasets.
(TXT)

S1 Text MSUPRP samples. This file contains a description of sample preparation, sequencing design and bioinformatics tools used for mapping reads and obtaining the count matrix for MSUPRP experiment.
(PDF)

S2 Text MSUPRP plasmodes. This file presents figures describing the plasmode generation process as well as the calculation of reference dissimilarity and reference dendrogram for MSUPRP plasmodes.
(PDF)

## References

[1] Liu P, Si Y. Cluster Analysis of RNA-Sequencing Data In: Datta S, Nettleton D, editors. Statistical Analysis of Next Generation Sequencing Data SE—10. Springer International Publishing; 2014 pp. 191–217. 10.1007/978-3-319-07212-8_10

[2] Jiang D, Tang C, Zhang A. Cluster analysis for gene expression data: a survey. IEEE Trans Knowl Data Eng. 2004;16: 1370–1386. 10.1109/TKDE.2004.68

[3] Dalton L, Ballarin V, Brun M. Clustering algorithms: on learning, validation, performance, and applications to genomics. Curr Genomics. 2009;10: 430–45. 10.2174/138920209789177601 20190957 PMC2766793

[4] Wang Z, Gerstein M, Snyder M. RNA-Seq: a revolutionary tool for transcriptomics. Nat Rev Genet. Nature Publishing Group; 2009;10: 57–63. 10.1038/nrg2484 PMC2949280 19015660

[5] Bullard JH, Purdom E, Hansen KD, Dudoit S. Evaluation of statistical methods for normalization and differential expression in mRNA-Seq experiments. BMC Bioinformatics. 2010;11: 94 10.1186/1471-2105-11-94 20167110 PMC2838869

[6] Law CW, Chen Y, Shi W, Smyth GK. Voom: precision weights unlock linear model analysis tools for RNA-seq read counts. Genome Biol. 2014;15: R29 10.1186/gb-2014-15-2-r29 24485249 PMC4053721

[7] Marioni JC, Mason CE, Mane SM, Stephens M, Gilad Y. RNA-seq: an assessment of technical reproducibility and comparison with gene expression arrays. Genome Res. 2008;18: 1509–1517. 10.1101/gr.079558.108 18550803 PMC2527709

[8] Robinson MD, McCarthy DJ, Smyth GK. edgeR: a Bioconductor package for differential expression analysis of digital gene expression data. Bioinformatics. 2009/11/17 ed. 2010;26: 139–140. 10.1093/bioinformatics/btp616 19910308 PMC2796818

[9] Anders S, Huber W. Differential expression analysis for sequence count data. Genome Biol. 2010/10/29 ed. 2010;11: R106 10.1186/gb-2010-11-10-r106 20979621 PMC3218662

[10] Johnson RA, Wichern DW. Applied multivariate statistical analysis. 5th ed Upper Saddle River, N.J.: Prentice Hall; 2002.

[11] Love MI, Huber W, Anders S. Moderated estimation of fold change and dispersion for RNA-Seq data with DESeq2. Genome Biol. 2014;15: 550 10.1186/s13059-014-0550-8 25516281 PMC4302049

[12] Witten DM. Classification and clustering of sequencing data using a Poisson model. Ann Appl Stat. The Institute of Mathematical Statistics; 2011;5: 2493–2518. 10.1214/11-AOAS493

[13] Handl J, Knowles J, Kell DB. Computational cluster validation in post-genomic data analysis. Bioinformatics. 2005;21: 3201–12. 10.1093/bioinformatics/bti517 15914541

[14] Sloutsky R, Jimenez N, Swamidass SJ, Naegle KM. Accounting for noise when clustering biological data. Brief Bioinform. 2013;14: 423–36. 10.1093/bib/bbs057 23063929

[15] Mehta T, Tanik M, Allison DB. Towards sound epistemological foundations of statistical methods for high-dimensional biology. Nat Genet. 2004/09/02 ed. 2004;36: 943–947. 10.1038/ng1422 15340433

[16] Reeb PD, Steibel JP. Evaluating statistical analysis models for RNA sequencing experiments. Front Genet. 2013;4: 1–9. 10.3389/fgene.2013.00178 PMC3775431 24062766

[17] Cattell RB, Jaspers J. General Plasmode No. 30-10-5-2 for factor analytic exercises and research. Multivariate Behav Res. 1967; 57.

[18] Waller NG, Underhill JM, Heather A. Multivariate Behavioral A Method for Generating Simulated Plasmodes and Artificial Test Clusters with User-Defined Shape, Size, and. 2010; 37–41. 10.1207/S15327906Mb340201 26753933

[19] Gadbury GL, Xiang Q, Yang L, Barnes S, Page GP, Allison DB. Evaluating statistical methods using plasmode data sets in the age of massive public databases: an illustration using false discovery rates. PLoS Genet. 2008/06/21 ed. 2008;4: e1000098 10.1371/journal.pgen.1000098 18566659 PMC2409977

[20] Steibel JP, Poletto R, Coussens PM, Rosa GJM. A powerful and flexible linear mixed model framework for the analysis of relative quantification RT-PCR data. Genomics. Elsevier Inc.; 2009;94: 146–52. 10.1016/j.ygeno.2009.04.008 19422910

[21] Bottomly D, Walter NAR, Hunter JE, Darakjian P, Kawane S, Buck KJ, et al Evaluating Gene Expression in C57BL/6J and DBA/2J Mouse Striatum Using RNA-Seq and Microarrays. PLoS One. Public Library of Science; 2011;6: e17820 10.1371/journal.pone.0017820 PMC3063777 21455293

[22] Frazee A, Langmead B, Leek J. ReCount: A multi-experiment resource of analysis-ready RNA-seq gene count datasets. BMC Bioinformatics. 2011;12: 449 10.1186/1471-2105-12-449 22087737 PMC3229291

[23] Steibel JP, Bates RO, Rosa GJM, Tempelman RJ, Rilington VD, Ragavendran A, et al Genome-wide linkage analysis of global gene expression in loin muscle tissue identifies candidate genes in pigs. PLoS One. 2011;6: e16766 10.1371/journal.pone.0016766 21346809 PMC3035619

[24] Steibel JP, Reeb PD, Ernst CW, Bates RO. Mapping cis and trans-acting eQTL in swine populations 10th WCGALP. Vancouver, Canada; 2014.

[25] Trapnell C, Pachter L, Salzberg SL. TopHat: discovering splice junctions with RNA-Seq. Bioinformatics. 2009;25: 1105–1111. 10.1093/bioinformatics/btp120 19289445 PMC2672628

[26] Trapnell C, Roberts A, Goff L, Pertea G, Kim D, Kelley DR, et al Differential gene and transcript expression analysis of RNA-seq experiments with TopHat and Cufflinks. Nat Protoc. Nature Publishing Group, a division of Macmillan Publishers Limited. All Rights Reserved.; 2012;7: 562–578. 10.1038/nprot.2012.016 PMC3334321 22383036

[27] Anders S, Pyl PT, Huber W. HTSeq A Python framework to work with high-throughput sequencing data. Bioinformatics. 2015;31: 166–169. 10.1093/bioinformatics/btu638 25260700 PMC4287950

[28] Mehta TS, Zakharkin SO, Gadbury GL, Allison DB. Epistemological issues in omics and high-dimensional biology: give the people what they want. Physiol Genomics. 2006;28: 24–32. 10.1152/physiolgenomics.00095.2006 16968808

[29] Izenman AJ. Modern Multivariate Statistical Techniques Regression, Classification, and Manifold Learning. New York, New York, USA: Springer; 2008.

[30] R Development Core Team. R: A language and environment for statistical computing. Vienna, Austria: R Foundation for Statistical Computing; 2014.

[31] Love MI, Huber W, Anders S. Moderated estimation of fold change and dispersion for RNA-Seq data with DESeq2. bioRxiv. 2014; 10.1101/002832 PMC4302049 25516281

[32] Halkidi M, Batistakis Y, Vazirgiannis M. On Clustering Validation Techniques. J Intell Inf Syst. 2001;17: 107–145.

[33] Xiong H, Li Z. Clustering Validation Measures Data Clustering. Chapman and Hall/CRC; 2013.

[34] Sokal RR, Rohlf FJ. The comparison of dendrograms by objective methods. Taxon. 1962;11: 33–40.

[35] Si Y, Liu P, Li P, Brutnell TP. Model-Based Clustering for RNA-Seq Data. Bioinformatics. 2014;30: 197–205. 10.1093/bioinformatics/btt632 24191069

[36] Rau A, Maugis-Rabusseau C, Celeux G. Co-expression analysis of high-throughput transcriptome sequencing data with Poisson mixture models. Bioinformatics. 2015;31: 1420–1427. 10.1093/bioinformatics/btu845 25563332

[37] Vaughan LK, Divers J, Padilla M, Redden DT, Tiwari HK, Pomp D, et al The use of plasmodes as a supplement to simulations: A simple example evaluating individual admixture estimation methodologies. Comput Stat Data Anal. 2010/02/18 ed. 2009;53: 1755–1766. 10.1016/j.csda.2008.02.032 20161321 PMC2678733

[38] Zhou X, Lindsay H, Robinson MD. Robustly detecting differential expression in RNA sequencing data using observation weights. Nucleic Acids Res. 2014; 1–10. 10.1093/nar/gku310 PMC4066750 24753412

[39] Ma C, Wang X. Application of the Gini correlation coefficient to infer regulatory relationships in transcriptome analysis. Plant Physiol. 2012;160: 192–203. 10.1104/pp.112.201962 22797655 PMC3440197

[40] Gadbury G, Garrett K, Allison D. Challenges and Approaches to Statistical Design and Inference in High-Dimensional Investigations In: Belostotsky DA, editor. Plant Systems Biology SE—9. Humana Press; 2009 pp. 181–206 LA—English. 10.1007/978-1-60327-563-7_9 PMC4667951 19588106

[41] Hastie T, Tibshirani R, Friedman JH. The Elements of Statistical Learning data Mining, Inference, and Prediction. Second Edi New York, New York, USA: Springer; 2009.

[42] Severin AJ, Woody JL, Bolon Y-T, Joseph B, Diers BW, Farmer AD, et al RNA-Seq Atlas of Glycine max: a guide to the soybean transcriptome. BMC Plant Biol. 2010;10: 160 10.1186/1471-2229-10-160 20687943 PMC3017786

[43] Dillies MA, Rau A, Aubert J, Hennequet-Antier C, Jeanmougin M, Servant N, et al A comprehensive evaluation of normalization methods for Illumina high-throughput RNA sequencing data analysis. Brief Bioinform. 2012; 10.1093/bib/bbs046 22988256

[44] Robinson MD, Oshlack A. A scaling normalization method for differential expression analysis of RNA-seq data. Genome Biol. 2010/03/04 ed. 2010;11: R25 10.1186/gb-2010-11-3-r25 20196867 PMC2864565

[45] Kendall MG, Gobbons JD. Rank Correlation Methods 5th ed Science Forum. USA: Oxford University Press; 1990.

[46] Pachter L. Models for transcript quantification from RNA-Seq. ArXiv. 2011;1104.3889: 1–28.

[47] Rau A, Gallopin M, Celeux G, Jaffrézic F. Data-based filtering for replicated high-throughput transcriptome sequencing experiments. Bioinformatics. 2013;29: 2146–2152. 10.1093/bioinformatics/btt350 23821648 PMC3740625

[48] Bourgon R, Gentleman R, Huber W. Independent filtering increases detection power for high-throughput experiments. Proc Natl Acad Sci. 2010;107: 9546–9551. 10.1073/pnas.0914005107 20460310 PMC2906865

[49] Van Iterson M, Boer JM, Menezes RX. Filtering, FDR and power. BMC Bioinformatics. 2010/09/09 ed. 2010;11: 450 10.1186/1471-2105-11-450 20822518 PMC2949886

