# Supplementary material for: Assessing Dissimilarity Measures for Sample-Based Hierarchical Clustering of RNA Sequencing Data Using Plasmode Datasets
Source: PLoS One. 2015 Jul 10;10(7):e0132310. doi: 10.1371/journal.pone.0132310 (PMC4498680; doi:10.1371/journal.pone.0132310)
Supplement: S1 Text — This file contains a description of sample preparation, sequencing design and bioinformatics tools used for mapping reads and obtaining the count matrix for MSUPRP experiment. (PDF) [file pone.0132310.s002.pdf]

Supporting Information: S1 Text. **MSUPRP samples.** This file contains a description of sample preparation, sequencing design and bioinformatics tools used for mapping reads and obtaining the count matrix.

Assessing dissimilarity measures for hierarchical clustering of samples from RNA sequencing data using plasmode datasets

Pablo D. Reeb[1,2], Sergio J. Bramardi[2,3], Juan P. Steibel[1,4*]

[1]Department of Fisheries and Wildlife, Michigan State University, East Lansing, MI, USA
[2]Department of Statistics, Universidad Nacional del Comahue, Cinco Saltos, RN, Argentina
[3]College of Agricultural and Forest Sciences, Universidad Nacional de La Plata, La Plata, Bs. As., Argentina
[4]Department of Animal Science, Michigan State University, East Lansing, MI, USA
[*]Corresponding author

Email addresses: PDR: reebpabl@msu.edu; SJB: sbramardi@gmail.com; JPS: steibelj@msu.edu

Sample preparation, sequencing and mapping

The MSUPRP dataset corresponds to 24 samples of *longissimus* muscle extracted from 24 F2 females selected from the MSU Pig Resource Population [1]. Total RNA was obtained using TRIzol reagent (Invitrogen/Life Technologies, Carlsbad, CA, USA), and RNA quantity and quality were determined using an Agilent 2100 Bioanalyzer (RIN ≥ 7). RNA was reverse transcribed into cDNA, fragmented and labeled to generate 24 barcoded libraries that were sequenced on an Illumina HiSeq 2000 (100 bp, paired-end reads) at the Michigan State University Genomics Core Facility. Four technical replicates were collected from each library and arranged in four different lanes of a flowcell allowing up to 12 barcodes per lane as illustrated in Fig. S1.

lane 1	lane 2	lane 3	lane 4	lane 5	lane 6	lane 7	lane 8
1034	1116	1034	1116	1034	1116	1034	1116
1154	1512	1154	1512	1154	1512	1154	1512
1194	1502	1194	1502	1194	1502	1194	1502
1058	1594	1058	1426	1058	1426	1058	1426
1640	1134	1640	1134	1640	1134	1640	1134
1300	1580	1300	1580	1300	1580	1300	1580
1484	1662	1484	1662	1484	1662	1484	1662
1170	1096	1170	1096	1170	1096	1170	1096
1534	1080	1534	1080	1534	1080	1534	1080
1644	1458	1644	1458	1644	1458	1644	1458
1426	1278	1426	1278	1426	1278	1426	1278
1240	1434	1240	1434	1240	1434	1240	1434

Figure S1. Sequencing layout of 24 barcoded samples. Each library has a tag with the sample number and a colour symbolizing its barcode. A same set of 12 tags is repeated 4 times (technical replicates).

The raw read data consisted of 96 pairs of fastq files (4 per sample) containing approximately 15 million short-reads (100bp) each. Those fastq files were pre-processed using FASTX toolkit (http://hannonlab.cshl.edu/fastx_toolkit/) and FASTQC (http://www.bioinformatics.babraham.ac.uk/projects/fastqc/) to assess read quality. Then, Tophat [2] was used for mapping the reads to the reference genome (Sus scrofa 10.2.69 retrieved from the Ensembl database) using an index generated by Bowtie2 [3]. The aligned records were stored in BAM/SAM format [4]. Alignment statistics and base coverage was calculated for each file using SAMTools [4]. After that, Cufflinks software [5] was used to obtain gene models and to merge gene models from all samples and reference annotation. Finally, transcript specific read counts were estimated using HTSeq[6].

Consistently, about 85% of reads were successfully mapped to reference genome. We detected a total of 26740 transcripts with at least one read aligned. Average coverage per base across 96 pairs of fastq files was 45.79X.

To obtain the final count matrix, we filtered out transcript with zero expression in all samples and merged the 4 technical replicates from each sample. As a result we obtain a count matrix with 26740 transcripts and 24 samples.

References

1. Steibel JP, Bates RO, Rosa GJM, Tempelman RJ, Rilington VD, et al. (2011) Genome-wide linkage analysis of global gene expression in loin muscle tissue identifies candidate genes in pigs. PLoS One 6: e16766. doi:10.1371/journal.pone.0016766.

2. Trapnell C, Pachter L, Salzberg SL (2009) TopHat: discovering splice junctions with RNA-Seq. Bioinformatics 25: 1105–1111.

3. Langmead B, Salzberg SL (2012) Fast gapped-read alignment with Bowtie 2. Nat Methods 9: 357–360. doi:10.1038/nmeth.1923.

4. Li H, Handsaker B, Wysoker A, Fennell T, Ruan J, et al. (2009) The Sequence Alignment / Map (SAM) Format and SAMtools 1000 Genome Project Data Processing Subgroup: 1–2.

5. Trapnell C, Roberts A, Goff L, Pertea G, Kim D, et al. (2012) Differential gene and transcript expression analysis of RNA-seq experiments with TopHat and Cufflinks. Nat Protoc 7: 562–578. doi:10.1038/nprot.2012.016.

6. Anders S, Pyl PT, Huber W (2014) HTSeq - A Python framework to work with high-throughput sequencing data. bioRxiv: 0–4. doi:10.1101/002824.
