# Supplementary material for: Assessing Dissimilarity Measures for Sample-Based Hierarchical Clustering of RNA Sequencing Data Using Plasmode Datasets
Source: PLoS One. 2015 Jul 10;10(7):e0132310. doi: 10.1371/journal.pone.0132310 (PMC4498680; doi:10.1371/journal.pone.0132310)
Supplement: S2 Text — This file presents figures describing the plasmode generation process as well as the calculation of reference dissimilarity and reference dendrogram for MSUPRP plasmodes. (PDF) [file pone.0132310.s003.pdf]

Supporting Information: S2 Text. MSUPRP plasmodes. This file presents figures describing the plasmode generation process as well as the calculation of reference dissimilarity matrix and reference dendrogram for MSUPRP plasmodes

Assessing dissimilarity measures for hierarchical clustering of samples from RNA sequencing data using plasmode datasets

Pablo D. Reeb[1,2], Sergio J. Bramardi[2,3], Juan P. Steibel[1,4*]

[1]Department of Fisheries and Wildlife, Michigan State University, East Lansing, MI, USA
[2]Department of Statistics, Universidad Nacional del Comahue, Cinco Saltos, RN, Argentina
[3]College of Agricultural and Forest Sciences, Universidad Nacional de La Plata, La Plata, Bs. As., Argentina
[4]Department of Animal Science, Michigan State University, East Lansing, MI, USA
[*]Corresponding author

Email addresses: PDR: reebpabl@msu.edu; SJB: sbramardi@gmail.com; JPS: steibelj@msu.edu

Plasmode generation for MSUPRP samples

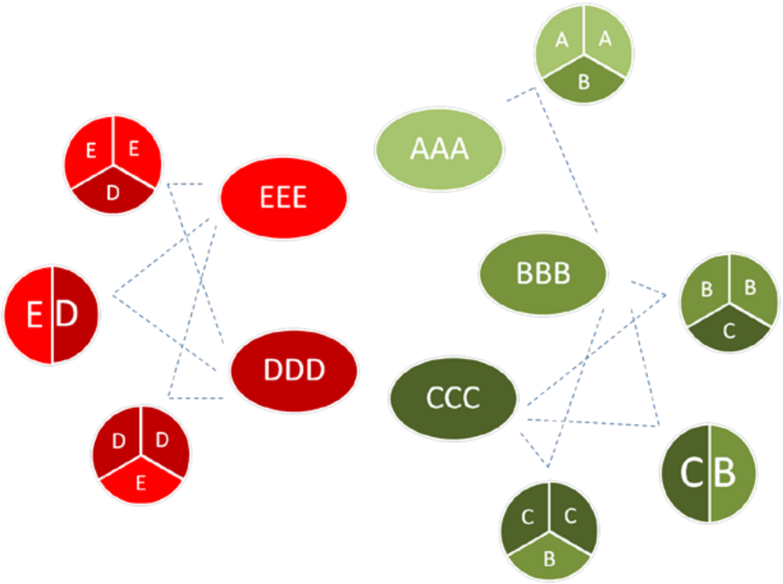

Figure S2. Plasmode generation for MSUPRP dataset. A singular plasmode dataset comprised 12 samples: 5 original samples, ovals labelled as {AAA,BBB,CCC,DDD,EEE}, and 7 synthetic samples, circles labelled as {AAC,BBC,CXB,CCB,DDE,EXD,EED}, obtained by combining known proportions of transcripts (½ , ⅓ , or ⅔) from the original samples.

Reference similarity and dissimilarity matrices for MSUPRP plasmodes

	AAA	AAC	BBB	BBC	CXB	CCB	CCC	DDD	DDE	EXD	EED	EEE
AAA	1.00	0.66	0.00	0.00	0.00	0.00	0.00	0.00	0.00	0.00	0.00	0.00
AAC	0.66	1.00	0.00	0.33	0.33	0.33	0.33	0.00	0.00	0.00	0.00	0.00
BBB	0.00	0.00	1.00	0.66	0.50	0.33	0.00	0.00	0.00	0.00	0.00	0.00
BBC	0.00	0.33	0.66	1.00	0.83	0.66	0.33	0.00	0.00	0.00	0.00	0.00
CXB	0.00	0.33	0.50	0.83	1.00	0.83	0.50	0.00	0.00	0.00	0.00	0.00
CCB	0.00	0.33	0.33	0.66	0.83	1.00	0.66	0.00	0.00	0.00	0.00	0.00
CCC	0.00	0.33	0.00	0.33	0.50	0.66	1.00	0.00	0.00	0.00	0.00	0.00
DDD	0.00	0.00	0.00	0.00	0.00	0.00	0.00	1.00	0.66	0.50	0.30	0.00
DDE	0.00	0.00	0.00	0.00	0.00	0.00	0.00	0.66	1.00	0.83	0.66	0.33
EXD	0.00	0.00	0.00	0.00	0.00	0.00	0.00	0.50	0.83	1.00	0.83	0.50
EED	0.00	0.00	0.00	0.00	0.00	0.00	0.00	0.30	0.66	0.83	1.00	0.66
EEE	0.00	0.00	0.00	0.00	0.00	0.00	0.00	0.00	0.33	0.50	0.66	1.00

Figure S3. Reference similarity matrix (S) for MSUPRP plasmodes. The similarity between two samples (s_{ij}) was calculated as the maximum proportion of original shared reads**.**

	AAA	AAC	BBB	BBC	CXB	CCB	CCC	DDD	DDE	EXD	EED
AAC	0.34										
BBB	1.00	1.00									
BBC	1.00	0.67	0.34								
CXB	1.00	0.67	0.50	0.17							
CCB	1.00	0.67	0.67	0.34	0.17						
CCC	1.00	0.67	1.00	0.67	0.50	0.34					
DDD	1.00	1.00	1.00	1.00	1.00	1.00	1.00				
DDE	1.00	1.00	1.00	1.00	1.00	1.00	1.00	0.34			
EXD	1.00	1.00	1.00	1.00	1.00	1.00	1.00	0.50	0.17		
EED	1.00	1.00	1.00	1.00	1.00	1.00	1.00	0.70	0.34	0.17	
EEE	1.00	1.00	1.00	1.00	1.00	1.00	1.00	1.00	0.67	0.50	0.34

Figure S4. Reference dissimilarity matrix (D) for MSUPRP plasmodes. The dissimilarity between two samples (d_{ij}) was calculated as 1- s_{ij} **.**

Reference dendrogram and cophenetic matrix for MSUPRP plasmodes

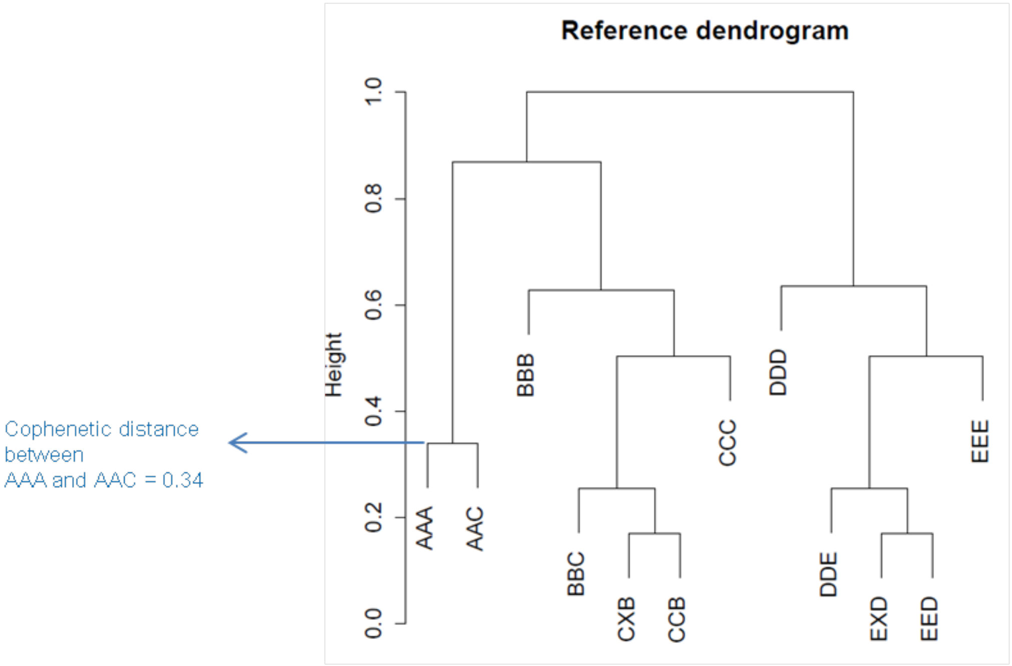

Figure S5. Reference dendrogram for MSUPRP plasmodes.

	AAA	AAC	BBB	BBC	CXB	CCB	CCC	DDD	DDE	EXD	EED
AAC	0.34										
BBB	0.87	0.87									
BBC	0.87	0.87	0.63								
CXB	0.87	0.87	0.63	0.26							
CCB	0.87	0.87	0.63	0.26	0.17						
CCC	0.87	0.87	0.63	0.50	0.50	0.50					
DDD	1.00	1.00	1.00	1.00	1.00	1.00	1.00				
DDE	1.00	1.00	1.00	1.00	1.00	1.00	1.00	0.64			
EXD	1.00	1.00	1.00	1.00	1.00	1.00	1.00	0.64	0.26		
EED	1.00	1.00	1.00	1.00	1.00	1.00	1.00	0.64	0.26	0.17	
EEE	1.00	1.00	1.00	1.00	1.00	1.00	1.00	0.64	0.50	0.50	0.50

Figure S6. Cophenetic matrix of the reference dendrogram for MSUPRP plasmodes.

Correlation between dissimilarity measures and reference dissimilarity for the MSUPRP plasmodes

Dissimilarity	Correlation with reference dissimilarity
raw	0.57 (0.075)
rnr	0.53 (0.069)
rld	0.83 (0.001)
vsd	0.82 (0.001)
pea	0.51 (0.045)
plg	0.82 (0.001)
spe	0.81 (0.001)
poi	0.81 (0.01)

Table S1. Correlation between dissimilarity measures and reference dissimilarity. Mean and standard deviation of correlation between each of the eight dissimilarity matrices and the reference dissimilarity for MSUPRP plasmodes (N=50 plasmode datasets). Euclidean distances using raw count data (*raw*), Euclidean distances using normalized samples (*rnr*), Euclidean distances using regularized logarithm (*rld*), Euclidean distances using variance stabilizing transformation (*vsd*), 1- Pearson correlation using raw counts (*pea*), 1- Pearson correlation using counts transformed by logarithm (plg), and 1- Spearman correlation using raw counts (*spe*), and Poisson dissimilarity (*poi*). Distances measures *raw*, *rnr*, and *pea* poorly preserved the expected sample structure (r<0.57) while *poi*, *rld*, *vsd*, *plg*, and *spe* highly preserved the expected sample structure (r>0.8).
